# A CAD System for Lung Cancer Detection Using Hybrid Deep Learning Techniques

**DOI:** 10.3390/diagnostics13061174

**Published:** 2023-03-19

**Authors:** Ahmed A. Alsheikhy, Yahia Said, Tawfeeq Shawly, A. Khuzaim Alzahrani, Husam Lahza

**Affiliations:** 1 Department of Electrical Engineering, College of Engineering, Northern Border University, Arar 91431, Saudi Arabia; yahia.said@nbu.edu.sa; 2 Department of Electrical Engineering, Faculty of Engineering at Rabigh, King Abdulaziz University, Jeddah 21589, Saudi Arabia; tshawly@kau.edu.sa; 3 Department of Medical Laboratory Technology, Faculty of Applied Medical Sciences, Northern Border University, Arar 91431, Saudi Arabia; akaalz@nbu.edu.sa; 4 Department of Information Technology, College of Computing and Information Technology, King Abdulaziz University, Jeddah 21589, Saudi Arabia; hlahza@kau.edu.sa

**Keywords:** lung cancer, classification, diagnosis, DCNN, VGG-19, LSTMs, medical informatics, artificial intelligence, CAD

## Abstract

Lung cancer starts and spreads in the tissues of the lungs, more specifically, in the tissue that forms air passages. This cancer is reported as the leading cause of cancer deaths worldwide. In addition to being the most fatal, it is the most common type of cancer. Nearly 47,000 patients are diagnosed with it annually worldwide. This article proposes a fully automated and practical system to identify and classify lung cancer. This system aims to detect cancer in its early stage to save lives if possible or reduce the death rates. It involves a deep convolutional neural network (DCNN) technique, VGG-19, and another deep learning technique, long short-term memory networks (LSTMs). Both tools detect and classify lung cancers after being customized and integrated. Furthermore, image segmentation techniques are applied. This system is a type of computer-aided diagnosis (CAD). After several experiments on MATLAB were conducted, the results show that this system achieves more than 98.8% accuracy when using both tools together. Various schemes were developed to evaluate the considered disease. Three lung cancer datasets, downloaded from the Kaggle website and the LUNA16 grad challenge, were used to train the algorithm, test it, and prove its correctness. Lastly, a comparative evaluation between the proposed approach and some works from the literature is presented. This evaluation focuses on the four performance metrics: accuracy, recall, precision, and F-score. This system achieved an average of 99.42% accuracy and 99.76, 99.88, and 99.82% for recall, precision, and F-score, respectively, when VGG-19 was combined with LSTMs. In addition, the results of the comparison evaluation show that the proposed algorithm outperforms other methods and produces exquisite findings. This study concludes that this model can be deployed to aid and support physicians in diagnosing lung cancer correctly and accurately. This research reveals that the presented method has functionality, competence, and value among other implemented models.

## 1. Introduction

Artificial intelligence (AI) has been used in various applications, such as in educational, industrial, economic, and medical fields. In the medical field, AI can detect and predict diseases. Recently, researchers have turned their attention to using AI in genomes to save lives and provide solutions for numerous diseases.

According to the World Health Organization (WHO) and [1], lung cancer has been reported as a widespread global disease that occurs due to the uncontrolled growth of tissues [1,2]. There has been a significant increase in the death rate from lung cancer [1]. In 2020, the International Association of Cancer Society (IACS) reported that 235,760 patients were diagnosed with lung cancer [1]. In the same year, around 132,000 deaths were announced by the IACS [1].

Lungs are spongy organs in the body. They are responsible for intaking oxygen from inhalation and expending carbon dioxide through exhalation [1,2,3]. There are numerous signs and symptoms of lung cancer. These symptoms include, but are not limited to, persistent coughing, coughing blood, shortness of breath, wheezing, and pain in the bones and chest [1,2]. These symptoms typically appear when the disease is at an advanced stage as opposed to an early stage [1,3,4,5,6,7]. Several factors can lead to a patient’s increased risk of lung cancer, such as prolonged smoking, exposure to secondhand smoke [2], exposure to radiation, and a family history of lung cancer [1,3,6,7].

The disease is diagnosed using CT scans, sputum cytology, or biopsy, which involves taking out a sample of infected lung tissue. Treatment plans are chosen based on several considerations, such as the patient’s overall health, disease stage, and preference. A physician’s recommendation is often a patient’s best course of action. Typically, a treatment plan includes surgery, chemotherapy, and radiation [8].

In general, two types of lung cancer exist, and these types are as follows [1,8,9,10]:

1. Non-small cell lung cancer (NSCLC) is the most common variant, which grows and spreads slowly;
2. Small cell lung cancer (SCLC) is caused by smoking and spreads faster than NSCLC.

**Figure 1:**
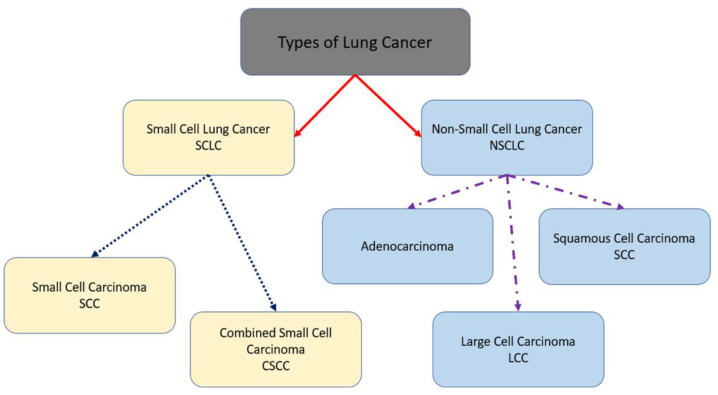
Types of lung cancer.

Adenocarcinoma, large cell carcinoma (LCC), and squamous cell carcinoma (SCC) are identified as the subtypes of NSCLC [11,12,13,14,15,16]. At the same time, small cell carcinoma and combined small cell carcinoma are classified as the subtypes of SCLC. Figure 1 depicts types of lung cancer. In this research, NSCLC is considered and studied.

In a recently published paper on lung cancer detection, the authors utilized circles inside the lungs to detect cancer. The circles were indicators of cancerous lungs. Herein, these circles are identified and classified using the proposed system.

### 1.1. Research Problem and Motivations

This article builds on a vision of Saudi 2030 to improve the quality of life of an individual society by developing a reliable approach to identifying lung cancer and providing accurate diagnoses that would save lives. The system offers healthcare providers an avenue for the comprehensive, integrated, and effective detection of lung cancer and the appropriate treatment administration to patients. Various studies have been conducted to identify and categorize the disease using numerous algorithms. The highest achieved accuracy was 99%, as in [3], while the F-score was 99.19%. Therefore, this study aims to achieve higher accuracy and F-score results.

The motivations of this research stem from the promising vision of Saudi Arabia, the 2030 vision. In 2016, the Saudi Arabia government announced and initiated this vision to start a new era. This era has become the dream for every citizen and resident in Saudi Arabia. The objectives of this vision are:

- Enhancing the economy of the country;
- Providing digital services and transforming public services into a digital world;
- Increasing safety and security;
- Providing a better life and improving the quality of life.

This vision involves 12 programs and initiatives, one of which is called the Quality-of-Life Program. It aims to create a better environment for Saudi citizens and residents by providing the necessary and possible lifestyle options. These options will support the government by engaging all people in the culture, sports, and activities inside the country. This engagement will increase social life, create more jobs, and improve the economy. In addition, utilizing and using new digital health solutions are critical in enhancing the quality of life. The authors of this research want to engage and take a place in this promising vision by proposing and developing a new approach that can elaborate and increase the current level of lung cancer diagnosis, identification, and analysis in Saudi Arabia.

### 1.2. Research Contributions

In this research, the contribution is achieved by proposing an automated, intelligent system to spot, identify, and classify NSCLC and its subtypes with high accuracy using two deep learning techniques. As found in the recently published articles in this field, as in [3], the current approaches detect and classify the tumors, and the maximum obtained accuracy was near 99%. Thus, the proposed system aims to close this gap and provides a bridge to enhance the identification and classification accuracy by implementing and developing a new model for lung cancer identification and categorization with a high accuracy, which was observed to be over 99.3%.

The rest of the paper is organized as follows: a literature review is presented in the next subsection, and Section 2 details the developing approach. The results and comparison evaluation between the developed method and other research in the literature are described in Section 3. The discussion is provided in Section 4, and the conclusion is given in Section 5.

### 1.3. Related Work

Hosseini et al. [1] provided a systematic review of lung cancer using deep learning approaches. The authors reviewed 32 conferences and journals related to lung cancer from 2016 to 2021 and combed through databases from IEEE Xplore, ScienceDirect, Springer Link, Wiley, and Google Scholar. Numerous algorithms for lung cancer detection were studied, evaluated, and analyzed based on their architecture, used datasets, and performance metrics, such as accuracy and sensitivity. Readers can find more information in [1].

Sousa et al. [2] analyzed a deep learning method developed on U-Net and ResNet34 structures. This procedure was performed on four different types of cross-cohort datasets. The authors evaluated the mean of a performance metric called the dice similarity coefficient (DSC) and found it to be more than 0.93. Two radiation experts spotted and determined the limitations of the developed method. The authors confirmed that there was a slight degradation for consolidation while testing two pathologist cases. In this article, the developed system reaches over 99.3% and 99.2% accuracy and F-scores, respectively. The presented algorithm evaluates the disease based on four performance metrics, as stated earlier. In addition, two deep learning tools were utilized to evaluate the system on three datasets. Interested readers can refer to [2] for additional information.

In [3], Nazir et al. proposed an approach to optimize lung cancer diagnosis using image fusion for segmentation purposes. This study incorporated and integrated the fusion method, designed and developed on a Laplacian pyramid (LP) decomposition with an adaptive sparse representation (ASR). This process worked because it fragmented the medical CT images into different sizes. At this point, the LP method was applied to fuse these sizes into four layers. A dataset was used to evaluate their approach using DSC as the performance metric, and it was nearly 0.9929. The authors claimed that their method produced better results than others recently published in the field. In contrast, the presented model uses three datasets and two deep learning techniques to achieve 99.42% accuracy, as the experiments show in Section 4. The algorithm reaches 99.76, 99.88, and 99.82% for recall, precision, and the F-score, respectively. This system developed three schemes and determined a confusion matrix for every scheme. These outcomes show that the proposed model is better than the developed method in [3] as it achieves higher outputs. Readers are advised to refer to [3] for additional information.

In [4], Dayma developed a manual process machine to detect lung cancer readily. This process involved numerous CT images and a Gabor filter, and a dataset of 1800 images, of which 900 were images of kids diagnosed with lung cancer. Each image was 200 × 200 pixels, and the dataset was collected from the IMBA Home Database. Unfortunately, there was no mention of any performance metric or obtained results. In contrast, the presented system is a CAD model, and three datasets were used to prove its flow and results. This CAD system achieves 99.42% average accuracy, and its maximum reached 99.61%. Moreover, the reached results of the other considered performance metrics were 99.76, 99.88, and 99.82% for recall, precision, and the F-score, respectively. Two deep learning techniques were used with three schemes to achieve these results. Both deep learning models were customized, as shown in the next section.

Hasan and Al Kabir [7] developed algorithms to determine whether cancer had spread in a patient’s lungs. These algorithms worked based on methods of image processing and statistical learning. The algorithms were tested on a dataset from the Kaggle site with 198 images. It achieved around 72.2% accuracy, which is significantly lower than the accuracy of the approach examined in this article, which is 99.42%. The proposed algorithm in this study achieves 99.76, 99.88, and 99.82% for recall, precision, and the F-score, respectively. These outcomes are exquisite and show that this method is better than the implemented one in [7]. Three scenarios were developed to reach acceptable results, as shown in Section 3. Moreover, the implemented method in [7] used one dataset with 198 images, while the presented system in this study only used three datasets with 1463 images for testing.

Nasser and Abu Naser [9] developed an artificial neural network (ANN) model to detect whether a human body contained lung cancer. This process involved numerous symptoms utilized as inputs to an ANN to diagnose the disease. Survey Lung Cancer, a dataset, was used to train and validate the model. It had an accuracy of nearly 96.67% after running more than 1,418,000 learning cycles. The approach took more time to reach this level of accuracy than the proposed approach in this article, which achieves more than 99% accuracy in fewer learning cycles. This difference demonstrates the superiority of the latter approach. Furthermore, compared to the model in [9], the execution time of this article’s approach is of a shorter duration. The CAD system achieved 99.42, 99.76, 99.88, and 99.82% for accuracy, recall, precision, and the F-score, respectively, using two deep learning tools, VGG-19 and LSTMs. These tools were customized and encapsulated, as illustrated in the next section. Readers are advised to refer to [9] for more information.

Bhatia et al. [15] implemented an algorithm for lung cancer detection using deep residual learning on CT-scan images. The authors used U-Net and ResNet models to extract features and highlight potential regions that are vulnerable to cancer. Multiple classifiers utilized to predict cancer included viz., XGBoost, RF, and individual predictions. This achieved 84% accuracy on an LIDC-IRDI dataset. In contrast, the presented system reached 99.42, 99.76, 99.88, and 99.82% for accuracy, recall, precision, and the F-score, respectively. This system utilized three scenarios using VGG-19 and LSTMs after performing various modifications. The obtained outcomes are better than the developed model in [15]. In addition, three datasets of CT scans and X-ray images were used.

Madan et al. [16] presented a technique to identify lung cancer using an ensemble CNN approach. This algorithm used CT scans and X-ray images of two datasets. This involved a dataset that contained 1623 images and achieved an accuracy of around 93%, lower than the obtained accuracy from the proposed CAD algorithm in this article. Conclusively, the proposed method in this article, for purposes of accuracy, achieves 99.42%, which is better than the results reached in [16]. Moreover, 99.76, 99.88, and 99.82% for recall, precision, and the F-score were also achieved. VGG-19 and LSTMs were modified to be utilized in this study.

## 2. Materials and Methods

### 2.1. Problem Statement

Healthcare providers need a solution that can give accurate diagnoses and results. The Ministry of Health in Saudi Arabia utilizes several devices to support and assist physicians in identifying lung cancer at an early stage with higher accuracy. Some implemented lung cancer diagnosis and classification methods that reached an accuracy between 98.8 and 99%, as in [2] and [3]. Hence, the authors target and intend to participate in the vision by providing a reliable solution to detect and classify lung cancer precisely with an accuracy higher than 99.3% and less processing time. The target processing time, also known as the execution time, is less than 3.5s for each input.

### 2.2. Research Objectives

This study aims to achieve numerous goals, and these objectives are summarized as follows:

- To explore, study, and analyze current methods in lung cancer to mark and locate their vulnerabilities;
- To search for available lung cancer datasets, download them, and conduct an analysis;
- To implement a feasible model to identify and categorize lung cancer by incorporating the convolutional neural network (VGG-19) and LSTMs;
- To determine the number of identified cancer cells using the developed model;
- To evaluate numerous performance parameters to assess the proposed algorithm with other state-of-the-art approaches using four parameters: accuracy, precision, recall, and the F-score;
- To build a confusion matrix that characterizes how the proposed model categorizes a given test dataset appropriately and accurately. The proposed CAD system generally generates three confusion matrixes as three schemes are utilized.

### 2.3. Datasets

The utilized datasets were downloaded from the Kaggle website [17,18] and LUNA16 grand challenge [19]. These datasets were approximately 70.125 GB, with 2,351 images of CT scans and X-ray types. These images were divided into four categories: adenocarcinoma, large cell carcinoma, squamous cell carcinoma, and normal. In total, 888 images were reserved for training and validation purposes, while the testing dataset included 1463 images. For the training and validation dataset, each category contained 222 images. The total number of extracted features per image was 22, such as area, diameter, texture, and radius. Therefore, the proposed system extracted 51,722 features/characteristics for all utilized images. Table 1 illustrates complete details about the used datasets, which include the type of images, the type of dataset, the size, and the ground truth.

### 2.4. The Utilized Deep Learning Techniques (DLTs)

#### 2.4.1. VGG-19

This tool is a convolutional neural network with 19 deep layers. It is considered another variation of the VGG technique. Visual Geometry Group developed it; thus, it is known as VGG. VGG-19 contains 16 convolutional layers, 3 fully connected layers, 5 max-pool layers, and 1 soft-max layer. In addition, there are 19.6 billion FLOPs. This tool accepts images of 224 × 224. Rectified linear unit (ReLU) activates each hidden layer to stabilize the system and regulate its parameters. In addition, the inputs were normalized using a batch normalization method. Moreover, the Adam optimizer was included in this tool. The obtained final characteristics matrix was paved and flattened to be fed into the dense layers. The kernel size was set to 3 × 3. The size of the pooling filter in every hidden layer was 2 × 2 with four strides. Due to space limitations, the architecture of VGG-19 is omitted. Table 2 summarizes the utilized hyperparameters for this study’s three datasets.

#### 2.4.2. Long Short-Term Memory Networks (LSTMs)

Another encapsulated deep learning tool in the proposed CAD system is LSTMs. Tanh was used as an activation inside this tool. To make the customization and integration of VGG-19 and LSTMs smooth and possible, the values of the common hyperparameters remained changed. Table 3 lists the utilized hyperparameters of LSTMs in this study. The Adam optimizer is included in this network as well.

### 2.5. The Proposed Methodology

The proposed approach in this article can detect and classify lung cancer quickly and accurately. This approach involves incorporating and elaborating numerous stages to achieve every physician’s core goal: saving lives. The proposed algorithm contains DCNN, specifically VGG-19, and LSTMs to detect and classify the disease. The proposed model in this article identifies and classifies only NSCLC quickly and correctly. This model uses principal component analysis (PCA) to reduce and minimize the resulting error [20,21,22,23]. In this proposed model, we deliver rich detail along with figures and charts to explain some results and represent outputs from the presented algorithm that are generated and initiated by the implemented approach.

**Figure 2:**
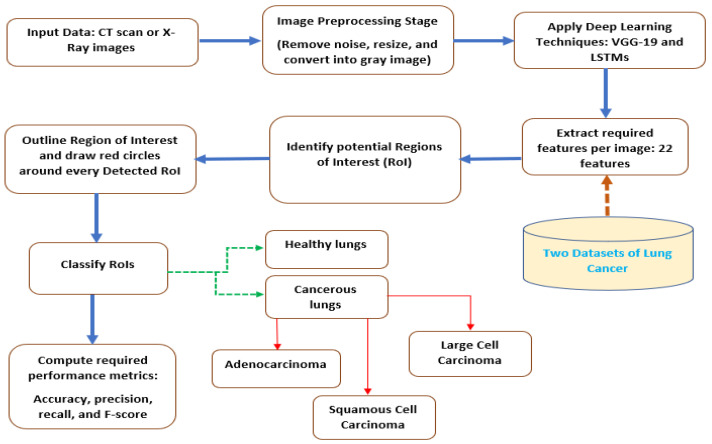
Flowchart of the proposed model.

In a recently published paper on lung cancer detection, the authors utilized circles inside the lungs to detect cancer. The circles were indicators of cancerous lungs. Here, the proposed algorithm uses DCNN and LSTMs after modifying them to detect lung cancer nodules and classify them. Moreover, it can help radiologists to provide efficient pattern recognition. Figure 2 depicts a block diagram of the presented model to identify and classify lung cancer nodules using three schemes of the considered tools. The used datasets from the Kaggle website and LUNA16 grad challenge are investigated to see how the presented approach works and responds to numerous procedures and operations.

The model starts by preprocessing to remove noise, resize the inputs, and convert all inputs into gray images. Gabor filters and a discrete wavelet transformation method distinguish lung regions and separate these regions from the original images.

The second stage of the proposed model is the deep learning phase, where VGG-19 and LSTMs are ensembled to identify and categorize the disease accordingly. Initially, the outputs from the previous step are normalized to support the training and testing stages, and all incoming data are resized to 224 × 224. Convolutional layers are required to generate the relevant features and map them according to the input information. Each filter in the convolutional layers contains a determined number of parameters that can learn through the training phase. These layers produce output features that are smaller than the input data. The number of utilized filters in the proposed model is 16, and these filters have a dimension of 3 × 3 with a size of 55 × 55, as illustrated in Table 2. In the medical field, the most utilized pool is the max-pool technique. Thus, it is adopted and modified in this research to extract features from every block of the characteristics maps. The proposed approach extracts numerous features to learn by itself. The total number of extracted features is 22 per image, including the area of the detected RoIs, diameter, standard deviation, and mean. The proposed CAD system extracts 51,722 features for all images. The size of the extracted features is reduced in the max pooling phase using principal component analysis (PCA) method. After generating the outputs of the extracted features, a sample of the training images is fed into the model to improve the accuracy of the profound learning results.

The next stage is identifying the potential RoIs according to the learned data and drawing red circles around all identified RoIs. This operation is performed in MATLAB using a built-in function. After that, the detected RoIs are classified according to the learned features using the utilized DLTs. The presented model classifies the disease into healthy, adenocarcinoma, squamous cell carcinoma, and large cell carcinoma, as illustrated in Figure 2. The extracted required characteristics are flattened and transformed into a one-dimensional array. Every image is normalized in every layer in the presented system. The multiplication of the generated matrices is carried in the fully connected layers. The soft-max layers distribute the extracted features into different groups. These groups are utilized to identify and classify the detected RoIs into their suitable classes, as depicted in Figure 2.

Finally, the proposed model evaluates numerous performance parameters to compare it with other state-of-the-art methods. The computed parameters are:

1. True Positive (TP): indicates the number of adequately identified types in the given dataset;
2. False Positive (FP): determines the number of types that are mispredicted;
3. True Negative (TN): gives the number of healthy lungs identified correctly;
4. False Negative (FN): measures the number of negative samples identified incorrectly;
5. Precision (PR): shows the ratio of the identified types over the summation of the classes that are identified incorrectly plus the actual classes that are correctly classified, as demonstrated in the equation below:
  PR = TP/(TP + FP) (1)
6. Recall (RE): computes the ratio of the identified classes over the summation of the actual images plus the number of negative types that are incorrectly classified, as depicted in (2):
  RE = TP/(TP + FN)(2)
7. Accuracy (Acc): this parameter indicates how the proposed approach performs well, and it is evaluated as follows:
  Acc = (TP + TN)/N(3)

where N is the total number of images being tested and computed as follows:

N = TP + TN + FN + FP(4)

8. F-score: represents a harmonic mean of two performance metrics of the presented CAD algorithm: recall and precision. Therefore, the higher the value is, the better the model is developed. This metric is evaluated as follows:
  F-score = 2 × [(PR × RE)/(PR + RE)](5)

The implemented model herein has various advantages. These advantages are as follows:

- It is easy to run and operate;
- Procedures are automated to minimize human intervention;
- It is a dependable and practical solution;
- No specific modules are mandatory.

The proposed Algorithm 1 to detect and classify lung cancer is illustrated as follows:
**Algorithm: Lung Cancer Detection and Classification**Input: an image: CT Scan or X-ray. Output: the detection and classification of Lung Cancer: NSCLC and its subtypes. Read an image or a sequence of images from a file.In the preprocessing phase: Do the following:Remove any detected noise.Rescale the inputs to the required size of VGG-19 and LSTMs.Apply various filters to enhance the pixels of the inputs.Transform the resultant image into a gray image.End of Preprocessing phase.For the Deep Learning phase: Do the following:Create a Zero matrix with a size = size of the input image by 4.For i =1: 4Perform a filtration: Gabor Filter and DWT to determine the magnitude and wavelength for every pixel.Perform a masking operation using the morphological process to extract the required features, such as Area, shape, diameter, and correlation.Determine a dynamic threshold for every image.Invert the image to separate the foreground and the background.Create a Binary image to detect and classify the disease with a size = 1024 × 1024.Find any potential area and draw a circle around it.Determine the number of detected areas and their drawn circles.For i = 1: 1024For j = 1: 1024Compute the number of white pixels z to compare it with the threshold.Plot the detected circles.If z > threshold:Cancer is Detected.EndClassify the detected cancer as Adenocarcinoma, LCC, or SCC.EndEnd of Deep Learning phase.Calculate the required performance parameters: accuracy, precision, recall, and F-score.End of the algorithm.

## 3. Results

Numerous simulation evaluation experiments were performed to verify the proposed approach and test its functionality and outputs. Several scenarios were tested to illustrate how the system works to detect the type of lung cancer present. In addition, the computation of performance parameters is presented as well. All simulation tests were carried out on a Microsoft Windows hosting machine. This hosting machine was run by Windows 11 Pro as its operating system. In addition, the clock was 2.4 GHz and had 16 GB of RAM. A further provision in this article is the comparative assessment between some literary works and the developed approach. The use of MATLAB in all simulation scenarios was paramount as it possesses built-in tools for image processing purposes. These tools were utilized and employed in the developed method. The implemented model/algorithm was tested over 200 times, and it took between 6 and 8 h for the training phase to achieve its highest results. During the training stage, the for-loop instruction was set to run around 3000 times to permit the algorithm to learn deeply to reach and accomplish acceptable results. The proposed approach produced four subgraphs for its outputs of detection and classification. Moreover, determining the number of detected lung cancer areas was provided. This article offers only three scenarios/cases of cancerous lungs. These cases represent the subtypes of NSCLC; these scenarios are as follows:

### 3.1. Scenario 1: Adenocarcinoma

**Figure 3:**
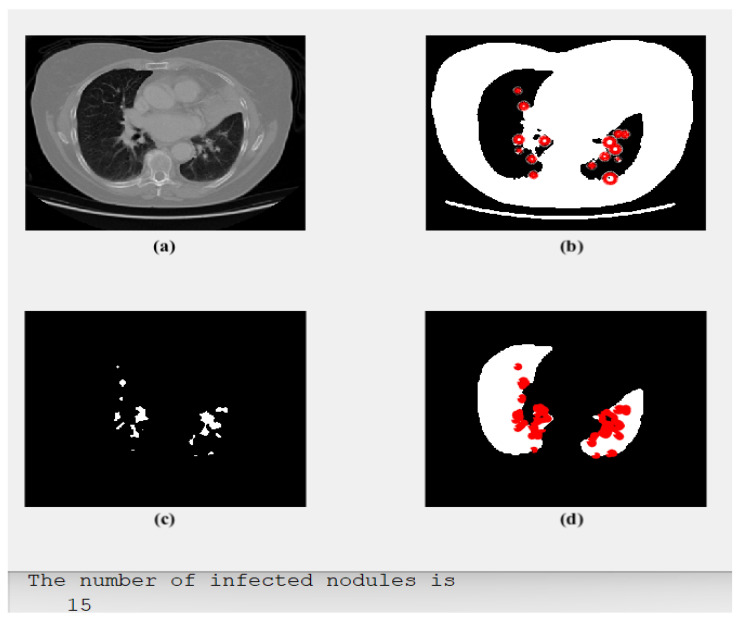
The obtained results of Case 1: (**a**) an input, (**b**) segmented potential RoIs, (**c**) the outlined spots, and (**d**) type of detected tumor: Adenocarcinoma.

Figure 3 illustrates four subgraphs: an original CT-Scan image in (a); (b) shows its segmented output with red circles around potential areas of possible cancer; (c) outlines only the detected regions or spots containing the disease; and (d) shows the cancerous lung with all detected cancer spots. Furthermore, the number of the detected nodules and their classification type are also offered.

As demonstrated and presented in Figure 3, the implemented model segmented the potential and possible regions of interest (RoIs) that could contain tumors. These regions of interest were determined by adding red circles, as illustrated in Figure 3b. Then, the proposed method outlined these regions of interest by removing all the other parts, as depicted in Figure 3c. Finally, these red circles/dots were restored to their original locations and placed in the original image, as in Figure 3d. The classification process result is shown in Figure 3d.

### 3.2. Scenario 2: Large Cell Carcinoma

**Figure 4:**
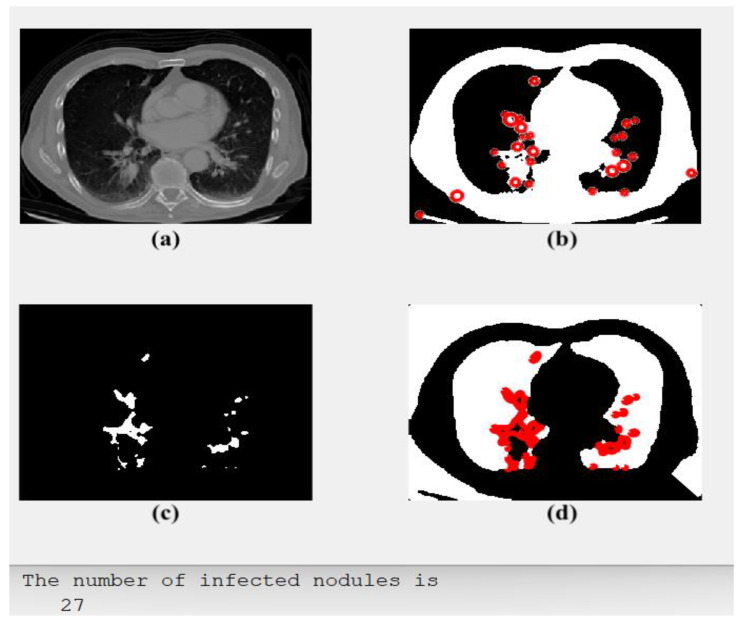
The obtained results of Case 2: (**a**) an input, (**b**) segmented potential RoIs, (**c**) the outlined spots, and (**d**) type of detected tumor: LCC.

Figure 4 illustrates an original CT-scan image in (a); (b) shows its segmented output with red circles around areas of possible cancer; (c) displays only the detected regions or spots containing the disease; and (d) shows the cancerous lung and the type of detected tumors. Furthermore, Figure 4 includes the computed number of detected cancer nodules and classification type.

The proposed CAD system generated the outputs, as shown in Figure 4. It discovers and learns by itself, through the deep learning phase, which type of discovered cancer has been identified. Each RoI is encircled in red, as depicted in Figure 4b, and all cancer spots are outlined and shown alone, as in Figure 4c. Moreover, the model verifies the number of all found cancer cells and their types, as in Figure 4d.

### 3.3. Scenario 3: Squamous Cell Carcinoma

**Figure 5:**
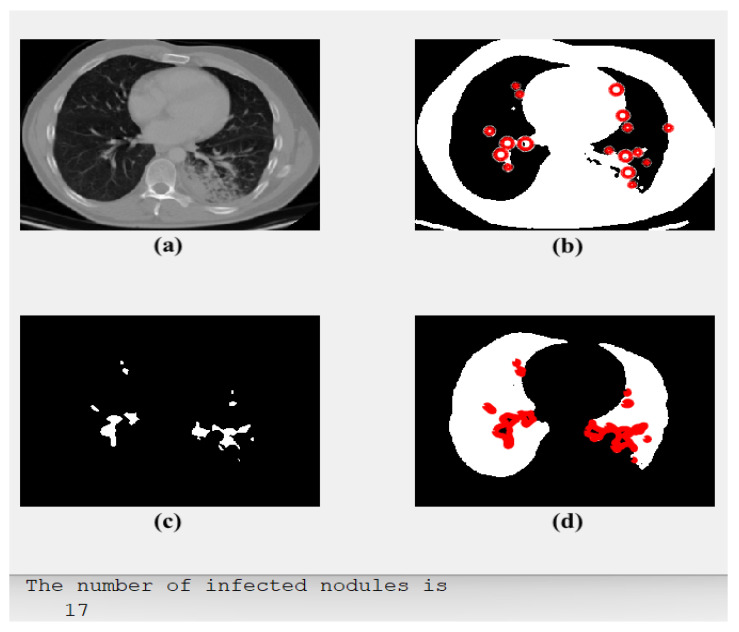
The results of Case 3: (**a**) an input, (**b**) segmented potential RoIs, (**c**) the outlined spots, and (**d**) type of detected tumor: SCC.

Figure 5 illustrates an original CT-scan image in (a); (b) shows its segmented output with red circles around areas of possible cancer; and (c) displays only the detected regions or spots. In addition, Figure 5 illustrates the estimated number of cancer spots the model identified and the result of the classification calculations.

Table 4, Table 5 and Table 6 list all the values of the performance parameters under consideration and measured by the proposed model for the three developed schemes. Table 4 represents the results of using VGG-19 alone, Table 5 shows the achieved results of LSTMs, while Table 6 demonstrates the outputs of combining VGG-19 and LSTMs. Accuracy, precision, recall, and F-scores are measured in percentages. In total, 850 CT-scan and X-ray images are shown. The developed algorithm reaches the highest outcomes of accuracy, precision, recall, and F-score, as shown in Table 6.

**Figure 6:**
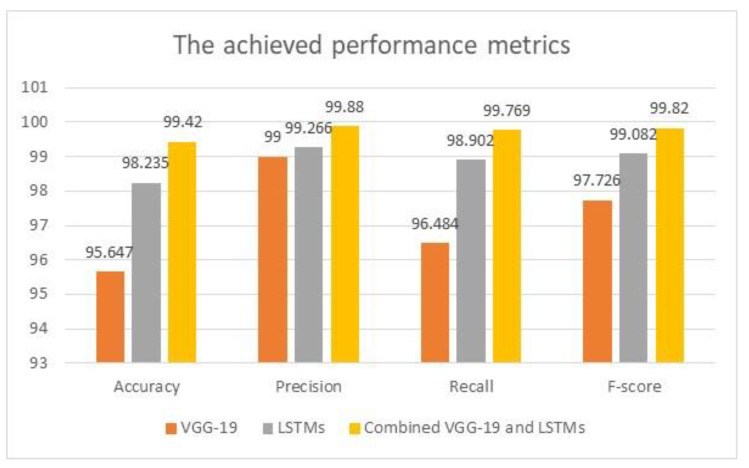
The evaluated performance metrics analysis.

The accuracy obtained increases to almost 99.61% when increasing the number of iterations inside the model and applying more testing inputs. Figure 6 depicts the graphical representation of the evaluated performance metrics of the three developed schemes.

Using the VGG-19 model standalone, the first scheme achieved the minimum results for all considered metrics, while the LSTMs reached better values. The last scheme, combining both techniques, achieved the highest outcomes for the considered metrics, as shown in Figure 6. Table 7 lists the comparison results for the average accuracy, precision, and recall values between the proposed algorithm and some research in the literature. Moreover, the utilized tools are included in Table 7, and N.M. stands for not mentioned. In this research, the obtained values of Table 6 are used in the comparison analysis.

Table 7 shows that the presented model generates better results than other works regarding all performance metrics. The implemented methods in [7,16] performed the lowest accuracy of 72.2 and 84%, respectively, while the works in [5,9] reached a moderate 93.548 and 96.67% accuracy, respectively. However, the proposed model displayed a maximum accuracy of 99.61%, which no other methods have achieved.

Table 8 demonstrates the obtained confusion matrix of the presented approach for 850 images of the third scheme, which gave the highest outcomes. The green marks the appropriate and correct categorized classes, while the red determines the improperly identified types. In addition, all subtypes of NSCLC are identified by the light orange color. Adenocarcinoma is Class A, Class B refers to LCC, Class C represents SCC, and Class D denotes healthy tissue.

**Figure 7:**
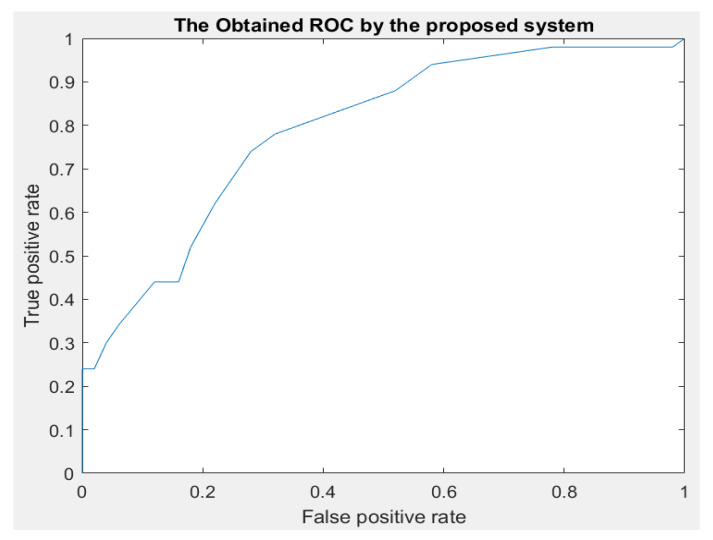
The evaluated performance metrics analysis.

**Figure 8:**
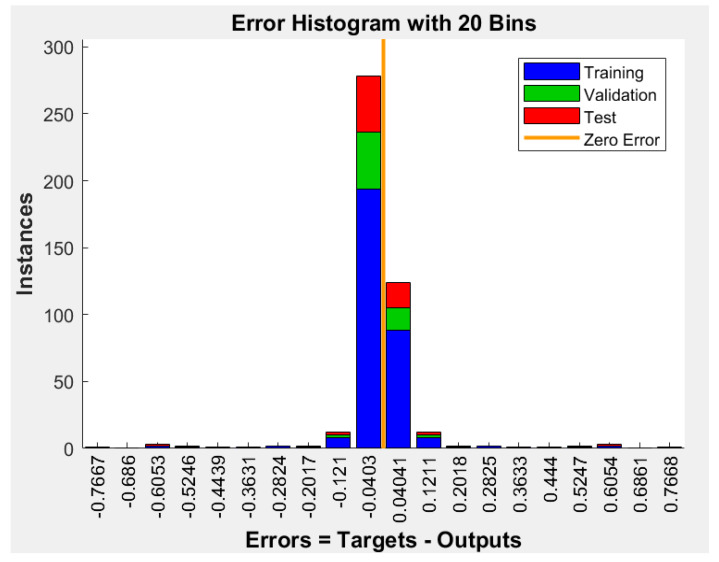
The achieved error histogram chart.

**Figure 9:**
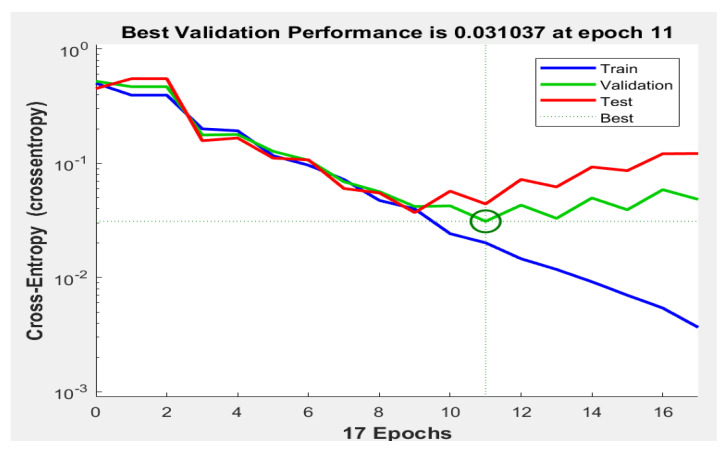
The achieved cross-entropy results.

Figure 7 and Figure 8 illustrate the achieved receiver operating characteristic (ROC) curve of the proposed system and the error histogram chart. Figure 7 shows the performance of classification by the system. Ten different thresholds were applied to obtain the classification results. In Figure 8, the error histogram was achieved with 20 bins. This figure includes the training, validation, and testing datasets. Figure 9 depicts the obtained cross-entropy results of the training, validation, and testing datasets. The best value of this quantity occurred at epoch 11.

## 4. Discussion

**Figure 10:**
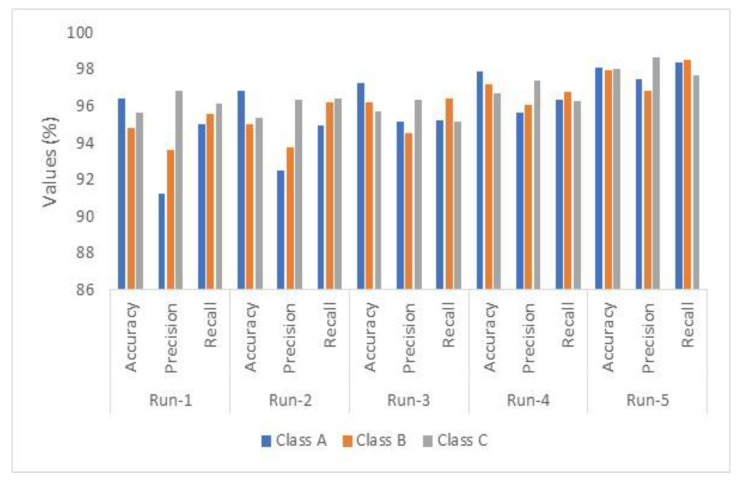
Result analysis of the proposed model.

The x-fold cross-validation technique was utilized in this research to evaluate the proposed model. This technique is a statistical method to estimate how the algorithm behaves and generates its outputs. In this article, five folds were conducted. Figure 10 represents the average graphical outputs for five different runs of the considered performance metrics: accuracy, precision, and recall of the third scheme after 150 iterations. In Figure 10, adenocarcinoma is called Class A, LCC is Class B, and SCC is Class C.

The proposed algorithm identifies and classifies NSCLC accurately, as demonstrated in the previous graphs. The implemented algorithm in this research and study integrates the VGG-19 tool with the LSTM technique to perform deep learning to diagnose and categorize lung tumors to reach and achieve the acceptable range of the considered performance parameters, as shown in Table 6. The presented CAD system surpasses other developed methods, as shown in Table 7, as no other methods have achieved the same results that were reached by the proposed system. The combination and integration of VGG-19 and LSTMs yielded the best and highest outcomes. Various steps and phases took place and were employed appropriately.

**Figure 11:**
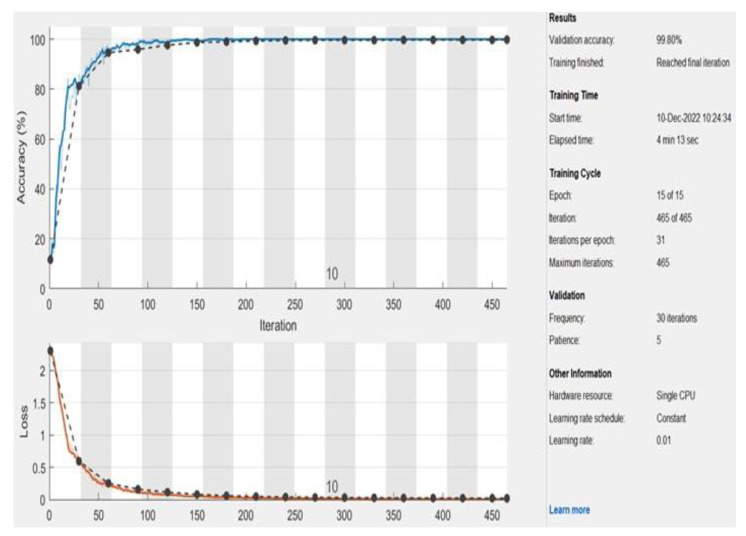
The achieved accuracy and loss function charts.

The executed and conducted evaluations on the achieved outcomes of the developed model show that it can distinguish, determine, and classify NSCLC correctly. Table 4, Table 5 and Table 6 list all model values when employing it on the 850 inputs. These inputs were CT scans and X-rays. The same tables detail the total number of inputs determined and correctly categorize the number of inputs classified inappropriately. Moreover, the comparative evaluation between the developed algorithm and its procedures and other state-of-the-art works is provided in Table 7. The resultant confusion matrix of the third scheme is represented in Table 8. This matrix shows that the presented system has the ability and the capability to identify and classify the disease correctly. These evaluations indicate and imply that the proposed model surpasses and outperforms other works in all performance metrics. Figure 11 illustrates the obtained accuracy and the loss function charts of the third scheme when the learning rate L was 0.01 for 15 epochs. This chart contains 465 iterations, with 31 iterations for each epoch. In addition, the validation occurs every 30 iterations. The black dashed lines refer to the validation process. The accuracy and the loss function become steady and stable after five epochs. The loss function converges nearly to 0, whereas the accuracy reaches 99.8%, as shown in the same graph.

**Figure 12:**
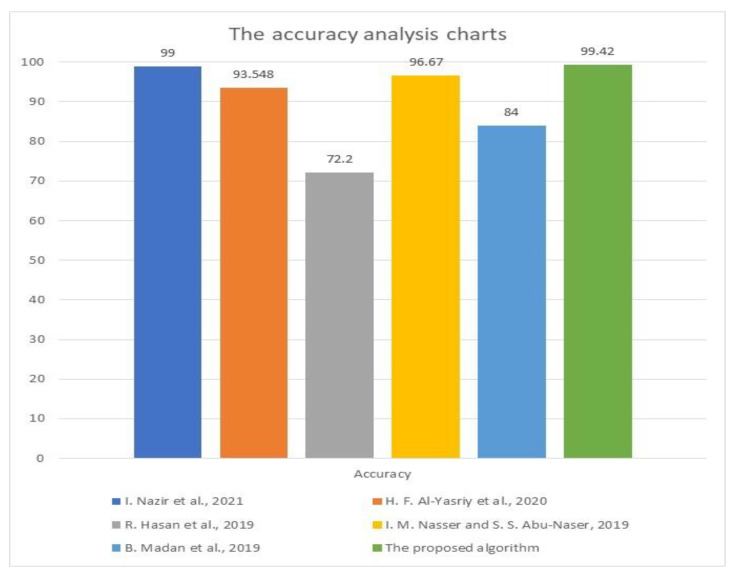
The comparative accuracy analysis charts between the proposed approach and some works [3,5,7,9,16].

Figure 12 illustrates the comparative accuracy analysis between the proposed system and some developed literature models. It shows that the presented model achieves the best accuracy results, and no other method could reach that accuracy level. The lowest achieved accuracy was in [7,16], while moderate values were obtained in [5,9]. The highest reached accuracy was in [3]. However, the proposed CAD method outperforms all these methods and achieved 99.42% on average, while its maximum result was 99.61%.

## 5. Conclusions

In this study, the developed model reveals a robust, trustworthy, and highly efficient system to detect and classify tumors from CT scans and X-ray images correctly and accurately. The implemented algorithm involves various tools, such as the Gabor filter, discrete wavelet transformation, PCA, and other filters, to deliver acceptable outcomes and results. This model possesses the capability and the ability to distinguish, differentiate, and classify tumors of the NSCLC types. The performance of the presented method was evaluated on the utilized three datasets from The Kaggle website and the LUNA16 grad challenge. From the attained findings, the implemented model surpasses all other methods regarding the considered metrics: accuracy, precision, and recall. The developed system generally shows considerable enhancements and enrichment on all considered metrics. The developed algorithm demonstrates its utility, efficiency, and accuracy in detecting and classifying NSCLC. Validation through MATLAB shows effective performance producing accepted outputs. The proposed algorithm reaches an average of 99.42% accuracy and around 99.61% when the number of iterations increases significantly. In addition, in some cases, the proposed system achieves 99.8% accuracy, and this is the only work that could reach this accuracy.

Future works are in place to improve the algorithm, leading to the appropriate classification of all subtypes and the production of accurate results from classification operations with minimal execution time.

## Figures and Tables

**Table 1:** The utilized datasets.

Properties	Chest CT Scans [17]	LIDC-IDRI [18]	LUNA16 [19]
Number of images	1000	463	888
Size	125 MB	4 GB	66 GB
Ground truth	Yes	Yes	Yes
Type	CT scan	X-rays	CT scan

**Table 2:** The hyperparameters of VGG-19.

Parameter	Used Value
Input size (n)	224 × 224
Activation (ACT)	ReLU
Kernel size (KS)	3 ×3
Pool filtering size (np)	2 × 2
Stride number (sn)	4
Padding (pad)	Same
Optimizer	Adam

**Table 3:** LSTMs’ hyperparameters and their values.

Parameter	Value
Number of cells (nc)	50
Number of units in every cell (nu)	64
Activation (ACT)	Tanh
Optimizer (opt)	Adam

**Table 4:** The evaluated performance metrics of VGG-19.

Performance Metric	Evaluated Value: N = 850 Images
TP	796 Adenocarcinoma = 245, LCC = 161, SCC = 233
TN	17
FN	29
FP	8
Accuracy	95.647%
Precision	99%
Recall	96.484%
F-score	97.726%

**Table 5:** The achieved outcomes of LSTMs.

Performance Metric	Evaluated Value: N = 850 Images
TP	811 Adenocarcinoma = 273, LCC = 201, SCC = 337
TN	24
FN	9
FP	6
Accuracy	98.235%
Precision	99.266%
Recall	98.902%
F-score	99.084%

**Table 6:** The evaluated performance analysis of combined VGG-19 and LSTMs.

Performance Metric	Evaluated Value: N = 850 Images
TP	833 Adenocarcinoma = 307, LCC = 289, SCC = 237
TN	11
FN	2
FP	1
Accuracy	99.42%%
Precision	99.880%
Recall	99.760%
F-score	99.820%

**Table 7:** Performance metrics and their values.

Works	Utilized Technology	Precision	Recall	Accuracy
Sousa et al., 2021 [2]	Fused image technique	N.M.	89%	99%
Nazir et al., 2021 [3]	LP + ASR	89%	N.M.	99%
Al-Yasriy et al., 2020 [5]	CNN	95.714%	N.M.	93.548%
Hasan et al., 2019 [7]	Image processing	N.M.	N.M.	72.2%
Nasser and Abu-Naser, 2019 [9]	ANN	N.M.	N.M.	96.67%
Madan et al., 2019 [16]	XGBoost + RFA	N.M.	N.M.	84%
The proposed algorithm	VGG-19 and LSTMs	99.42%	99.880%	99.760%

**Table 8:** The obtained confusion matrix of the third scheme.

**Predicted Class**	**True Class**				
		**Class A** **310**	**Class B** **289**	**Class C** **237**	**Class D** **14**
	Class A	307 = (99.032%)	0	0	1 = (7.143%)
	Class B	1 = (0.323%)	286 = (98.962%)	0	2 = (0.692%)
	Class C	0	0	237 = (100%)	0
	Class D	2 = (14.286%)	0	0	12 = (85.714%)

## Data Availability

The utilized datasets in this study were downloaded from the Kaggle website, and their links are available upon request.
